# Indoor Tanning in Europe: Temporal Trends, Sociodemographic Determinants from the Euromelanoma Campaign (2009–2018)

**DOI:** 10.3390/cancers18213548

**Published:** 2026-11-04

**Authors:** Aurora Gaeta, Mariano Suppa, Hassane Njimi, Nikolay Nikolaevich Potekaev, Alexander J. Stratigos, Alexander Katoulis, Åsa Ingvar, Ada Girnita, Olga Bogomolets, Jean-Luc Bulliard, Olivier Gaide, Juerg Hafner, Judith Oláh, Eszter Baltas, Vladimir Placek, Grażyna Kamińska-Winciorek, Lidija Kandolf, Liliana Medenica, Jana Třešňák Hercogová, Eduardo Nagore, Thomas Maselis, Osvaldo Correia, Ana Filipa Duarte, Hana Helppikangas, Michael Reusch, Alin Codrut Nicolescu, Ana-Maria Forsea, Mirna Šitum, Matilda Bylaite-Bucinskiene, Lally Mekokishvili, Maia Datuashvili, Ingrid Roscher, Henrik Frank Lorentzen, Ketty Peris, Nahide Onsun, Raimonds Karls, Pille Konno, Zorica Zafirovik, Tanja Planinšek Ručigaj, Ana Benedicic, Michelle Murphy, Angela Hayrapetyan, Michael Hambardzumyan, Lawrence Scerri, Daniel Micallef, Aliaksandr M. Lukyanau, Andreas Pallouras, Konstantinou Paschalis, Boris Nedelciuc, Wiam Al Bouzidi, Sara Gandini, Véronique del Marmol

**Affiliations:** 1 Department of Experimental Oncology, IEO European Institute of Oncology IRCCS, 20141 Milan, Italy; auroragaeta.epi@gmail.com; 2 Department of Statistics and Quantitative Methods, University of Milano-Bicocca, 20126 Milan, Italy; 3 Department of Dermatology, Hôpital Erasme (HUB), Université Libre de Bruxelles, 1070 Brussels, Belgium; 4 Department of Dermatology, Institut Jules Bordet (HUB), Université Libre de Bruxelles, 1070 Brussels, Belgium; 5 Biomedical Statistics, Université Libre de Bruxelles, 1070 Brussels, Belgium; 6 Moscow Scientific and Practical Center of Dermatovenereology and Cosmetology, 119071 Moscow, Russia; 7 First Department of Dermatology, National and Kapodistrian University of Athens School of Medicine, Andreas Sygros Hospital, 16121 Athens, Greece; 8 Second Department of Dermatology and Venereology, National and Kapodistrian University of Athens Medical School, Attikon University Hospital, 12462 Athens, Greece; alexanderkatoulis@yahoo.co.uk; 9 Department of Clinical Sciences, Lund University, 221 85 Lund, Sweden; asa.ingvar@gmail.com; 10 Department of Dermatology, Skåne University Hospital, 221 85 Lund, Sweden; 11 Skin Cancer Center, Theme Cancer, Karolinska University Hospital, 171 76 Stockholm, Sweden; 12 Institute of Dermatology and Cosmetology, 01024 Kyiv, Ukraine; 13 Center for Primary Care and Public Health (Unisanté), University of Lausanne, 1010 Lausanne, Switzerland; 14 Department of Dermatology and Venereology, Lausanne University Hospital (CHUV), 1011 Lausanne, Switzerland; 15 Department of Dermatology, University Hospital Zurich, 8091 Zurich, Switzerland; 16 Department of Dermatology and Allergology, University of Szeged, 6720 Szeged, Hungary; 17 Department of Dermatology, Sexually Transmitted Diseases and Clinical Immunology, University of Warmia and Mazury, 10-229 Olsztyn, Poland; 18 Department of Bone Marrow Transplantation and Onco-Hematology, Maria Skłodowska-Curie National Research Institute of Oncology, Gliwice Branch, 44-102 Gliwice, Poland; 19 Department of Dermatology, Faculty of Medicine, Military Medical Academy, 11040 Belgrade, Serbia; lkandolfsekulovic@gmail.com; 20 Department of Dermatology and Venereology, School of Medicine, University of Belgrade, 11000 Belgrade, Serbia; 21 Dermatology Prof. Hercogova, Chlumcanskeho 5, 180 00 Praha, Czech Republic; 22 Department of Dermatology, Instituto Valenciano de Oncología, 46009 Valencia, Spain; eduardo.nagore.e@gmail.com; 23 Private Practice, 3300 Tienen, Belgium; 24 Epidermis Dermatology Center, CUF Institute, 4460-188 Porto, Portugalduarte.af.t30@gmail.com (A.F.D.); 25 Portuguese Skin Cancer Association, 4430-196 Porto, Portugal; 26 Faculty of Medicine, University of Porto, 4200-319 Porto, Portugal; 27 Dermatology Department, Clinical Center, University of Sarajevo, 71000 Sarajevo, Bosnia and Herzegovina; 28 Dermatological Practice Tibarg, 22459 Hamburg, Germany; 29 Carol Davila University of Medicine and Pharmacy, 050474 Bucharest, Romania; nicolescualin66@yahoo.com; 30 Egoclinic, District 1, 010235 Bucharest, Romania; 31 Oncologic Dermatology Department, Elias University Hospital, Carol Davila University of Medicine and Pharmacy, 011461 Bucharest, Romania; aforsea@yahoo.com; 32 Department of Dermatovenereology, University Hospital ‘Sestre Milosrdnice’, 10000 Zagreb, Croatia; 33 Clinic of Infectious, Chest Diseases, Dermatovenereology and Allergology, Faculty of Medicine, Vilnius University, LT-08411 Vilnius, Lithuania; 34 Petre Shotadze Tbilisi Medical Academy, 0144 Tbilisi, Georgia; laliuniderma@yahoo.de; 35 Georgia Faculty of Medicine, Caucasus International University, 0192 Tbilisi, Georgia; maiaderma@gmail.com; 36 Department of Dermatology, Oslo University Hospital, 0424 Oslo, Norway; 37 Department of Dermatology, Aarhus University Hospital, 8200 Aarhus, Denmark; 38 Institute of Dermatology, Università Cattolica del Sacro Cuore, Fondazione Policlinico Universitario A. Gemelli IRCCS, 00168 Rome, Italy; ketty.peris@unicatt.it; 39 Department of Dermatology, School of Medicine, Bezmialem Vakif University, 34093 Istanbul, Turkey; 40 Derma Clinic Riga, Latvian University, LV-1007 Riga, Latvia; 41 Dermatology Outpatient Clinic, Clinic of Internal Medicine, East Tallinn Central Hospital, 10138 Tallinn, Estonia; 42 University Clinic of Dermatology, Faculty of Medicine, Ss. Cyril and Methodius University, 1000 Skopje, North Macedonia; drzoricazafirovik@gmail.com; 43 Dermatovenereological Clinic, University Medical Centre Ljubljana, 1000 Ljubljana, Slovenia; t.rucigaj@gmail.com; 44 Department of Dermatovenerology, University Medical Centre Maribor, 2000 Maribor, Slovenia; 45 Department of Dermatology, South Infirmary Victoria University Hospital, T12 X23H Cork, Ireland; 46 Department of Dermatology, Yerevan 0002, Armeniahambardzumyan.michael@gmail.com (M.H.); 47 Department of Dermatology and Venereology, Mater Dei Hospital, MSD 2090 Msida, Malta; scerri.lawrence@gmail.com (L.S.); daniel.b.micallef@gov.mt (D.M.); 48 Belarusian Medical Academy of Postgraduate Education (BelMAPE), 220013 Minsk, Belarus; 49 Cyprus Society of Dermatology and Venereology, 6023 Larnaca, Cyprus; 50 Department of Dermatovenerology, Nicolae Testemitanu State University of Medicine and Pharmacy, MD-2004 Chisinau, Moldova

**Keywords:** indoor tanning, sunbeds, melanoma prevention, skin cancer, Euromelanoma, Europe, risk factors, epidemiology, health behavior, gender

## Abstract

**Background:** In 2009, the International Agency for Research on Cancer (IARC) classified UV-emitting tanning devices as carcinogenic to humans (Group 1). This study aimed to describe ten-year trends in sunbed use prevalence across Europe and examine differences by country and sociodemographic and phenotypic characteristics. **Methods:** Euromelanoma survey data collected between 2009 and 2018 from 33 countries were analysed among individuals voluntarily attending free skin examinations. Annual sunbed use prevalence and 95% confidence intervals were estimated by country, with stratified analyses by gender, age, education level, and skin phototype. Associations with sunbed use were investigated using mixed-effects logistic regression models. **Results:** A total of 343,675 participants were included. Among Euromelanoma campaign participants, current sunbed use declined over the study period, declining from approximately 12.8% in 2009 to below 6% by 2018, despite year-to-year fluctuations. Women, adults aged 20–35 years and individuals with skin phototypes III–IV exhibited the highest odds of sunbed use (OR Female vs. Male = 3.01, 95%CI [2.91–3.12], OR 20–35 vs. >35 = 2.54, 95%CI [2.47–2.61], and OR Type III–IV vs. Type I–II = 1.52, 95%CI [1.47–1.56], respectively). Marked heterogeneity was observed across countries. Mixed-effects analyses showed a strong temporal decline that was significantly steeper among younger participants, particularly those aged 20–35 years, than among individuals older than 35 years. **Conclusions:** Indoor tanning prevalence declined across Europe between 2009 and 2018, during a period of evolving post-IARC regulatory and preventive measures. Persistent sociodemographic disparities and substantial between-country heterogeneity highlight the need for targeted, country-specific prevention strategies and continued monitoring of indoor tanning behaviors.

## Simple Summary

Indoor tanning is a well-established risk factor for skin cancer, yet little is known about how its use has changed across Europe over time. Using data from more than 340,000 participants in the Euromelanoma campaign from 33 European countries, we examined trends in sunbed use between 2009 and 2018 and identified population groups more likely to use indoor tanning devices. We found a substantial decline in sunbed use across Europe during the study period, particularly among younger adults. However, important differences persisted between countries and demographic groups. Women, individuals aged 20 to 35 years, and those with skin phototypes III to IV reported the highest prevalence of indoor tanning. These findings suggest that public health campaigns and regulatory measures may have helped reduce sunbed use, but continued prevention efforts remain necessary, especially in higher-risk populations and in countries where indoor tanning remains more common.

## 1. Background

In 2009, the International Agency for Research on Cancer (IARC) classified UV-emitting tanning devices as carcinogenic to humans (Group 1) [1]. Globally, cutaneous melanoma (CM) accounted for around 331,700 cancer cases in 2022 [2,3], while non-melanoma skin cancers (NMSCs), primarily basal cell carcinoma and squamous cell carcinoma, accounted for an estimated 1.23 million new cases and represent a particularly substantial burden in fair-skinned populations, with ultraviolet radiation being a major modifiable risk factor. About 80% of the CM burden is attributable to UVR [4]. Modern sunbeds emit high-intensity ultraviolet radiation (UVR), primarily UVA, often accompanied by small amounts of UVB, at levels not typically encountered in natural sunlight. These exposures can induce DNA damage in skin cells, as demonstrated in experimental studies [5]. Sunbed use is associated with a significant increase in the risk of melanoma. The risk increases with the number of sunbed sessions and with initial usage at a young age (<35 years), with an additional 1.8% extra risk per session per year [6,7,8]. Studies also show an association of sunbeds with an increased risk of basal cell carcinoma and squamous cell carcinoma, with dose–response relationships [7,8].

Usage in Europe is particularly common among young, urban women who engage in sun-seeking and other risk behaviors, and is more prevalent in northern, lower-sunshine countries, with some southern exceptions [8,9]. Italy and Spain have relatively high sunbed use despite higher sunshine, likely due to cultural/aesthetic norms [9]. In Europe, sunbed use is mainly driven by appearance-related motivations, such as the desire for an attractive look or a pre-vacation tan, as well as by social influences (Friends’, parents’, and peers’ tanning strongly normalize sunbed use) and perceived health benefits [10,11,12,13,14,15].

Available evidence indicates that sunbed use is particularly common among young adult women, individuals with Fitzpatrick skin phototype III–IV, and people with medium-to-high socioeconomic status. Sunbed users are also more likely to engage in other sun-seeking behaviours (e.g., sunbathing) and to report additional risk factors, including high nevus count, a history of sunburns, and smoking [9,13,14,16,17,18,19]. Tanning is most commonly accessed through tanning salons and beauty centers, but sunbeds are also available in sports facilities, hotels, and private settings. These factors should be considered when designing strategies to reduce artificial tanning, as users are often aware of its risks [20] but underestimate their personal susceptibility and believe that artificial tanning provides a healthy or protective base tan before natural sun exposure.

Over the past decade, indoor tanning practices and the regulatory landscape surrounding UV-emitting tanning devices have evolved, particularly following the classification of artificial tanning devices as carcinogenic to humans. International evidence suggests that the prevalence of indoor tanning has declined in several settings since the 2009 IARC classification and the subsequent expansion of indoor-tanning regulations, although the magnitude and consistency of these declines vary across populations and countries [21,22]. However, comparable longitudinal data spanning multiple European countries remain limited. Monitoring temporal patterns at the European level is important not only to document overall changes in sunbed use but also to determine whether declines have occurred consistently across population groups and countries and to identify subgroups in which sunbed use remains relatively common. Such information may help inform and refine targeted prevention strategies and support continued surveillance of indoor tanning behaviors.

Building on the previously published pan-European analysis of sunbed use [19], the present study addresses a complementary question by examining temporal patterns in self-reported current sunbed use over a ten-year period. Specifically, we assess whether these temporal trends differ across age and gender groups and characterise geographical heterogeneity across European countries. By using repeated cross-sectional data covering a substantially larger number of participants and applying multivariable mixed-effects models, the present analysis provides a longitudinal perspective on changes in current sunbed use that was not addressed in the previous report.

## 2. Methods

We conducted a repeated cross-sectional study using data collected during the Euromelanoma skin cancer prevention campaigns conducted across Europe between 2009 and 2018. The study period was restricted to 2009–2018 because comparable Euromelanoma data for subsequent years were unavailable for the present analysis. A new questionnaire was proposed in 2019, and Euromelanoma campaign-based data collection was subsequently disrupted by the COVID-19 pandemic [23]. These changes precluded the availability of a sufficiently comparable and continuous dataset for the present analysis.

The study included individuals who voluntarily attended free skin examinations. Participants completed a standardized self-administered questionnaire that gathered information on sociodemographic characteristics, sun exposure habits, and sunbed use [24]. Observations with missing values in any variable included in the analysis (outcome or covariates) were excluded.

The main outcome of interest was ‘current’ sunbed use, based on the question ‘Do you use solarium?’. A separate questionnaire item collected information on the number of years of sunbed use, including past use. Because the present study specifically aimed to investigate temporal trends in current sunbed use, current use was analysed as the primary outcome and was not combined with past use. The questionnaire did not specify a defined recall period for this question.

### 2.1. Variables

Age categories were defined a priori to distinguish adolescents, young adults, and older adults, and to facilitate comparison with previous Euromelanoma analyses considering the following categories: <20 years, 20–35 years, >35 years. Participants with missing age were excluded from the primary complete-case analysis and retained only in the missing-indicator sensitivity analysis. Educational level was classified as high (university degree or vocational education) or low (primary school or high school). Skin phototype was derived from self-reported skin reaction to sun exposure and grouped into: Type I–II (light skin) and Type III–IV (darker skin). For each country, we extracted the latitude of its capital city.

### 2.2. Statistical Analysis

Using counts and percentages, we compiled sociodemographic traits and the prevalence of sunbed use by country. To better understand year- and country-specific trends, the absolute change in the prevalence of current use from the previous year was calculated. Standard errors and 95% confidence intervals, computed using the Wald method, were included with the prevalence values. Given the opportunistic sampling design, the age, gender, education, and skin type composition of participants varied across countries and calendar years, potentially biasing crude prevalence comparisons. To assess associations between individual characteristics and sunbed use, we fitted a mixed-effects logistic regression model with a random intercept for country to account for between-country heterogeneity. Year-specific adjusted prevalence estimates were derived as marginal predicted probabilities from the fitted model. Fixed-effect predictors included gender, age group, educational level, skin phototype, and survey year.

Because missingness differed across countries, complete-case analysis was preferred to preserve consistency across multivariable models. As a sensitivity analysis, models were also fitted while retaining observations with missing characteristics, using an additional variable layer to capture their missingness. Prespecified interactions between survey year and gender and between survey year and age group were tested. For analyses of year-specific odds ratios, survey year was modeled as a categorical variable, with 2009 as the reference year. A decreasing pattern across years was interpreted as a reduction in sunbed use over time. For models assessing the average annual change in sunbed use and interaction with age and gender, survey year was entered as a continuous variable.

Country-year observations showing marked deviations from adjacent years were additionally checked against the original Euromelanoma database to identify potential data extraction or denominator inconsistencies. Such observations were retained when no evidence of data inconsistency was identified.

Odds ratios (ORs) and 95% confidence intervals were reported.

Adjusted prevalence estimates were obtained using a mixed-effects logistic regression model that included gender, age group, educational level, and skin phototype, with country as a random effect. Year-specific prevalence was derived as estimated marginal predicted probabilities (population-standardized predictions). The smoothing curves were fitted using a locally weighted regression approach, and 95% confidence intervals were displayed to illustrate the variability of the smoothed estimates.

LOESS smoothing was used to investigate the association between latitude and sunbed use prevalence across participating countries, allowing visualization of potential non-linear trends. version 4.5.1; R Foundation for Statistical Computing, Vienna, Austria), with the packages dplyr, gtsummary, tidyr, and ggplot2.

The Euromelanoma campaign collected anonymized data for research and surveillance purposes. No identifiable information was stored, and the study complied with applicable ethical standards.

## 3. Results

The study population increased from 227,888 in the previous report [19] to 410,937 participants and involved 33 countries; however, for some years and some countries, the information was incomplete. The final sample used for the model included 343,675 participants (83.6% of the study population). The number of observations collected for each year-country is reported in Figure S1.

Approximately 16.4% of observations were excluded during data cleaning, including those with missing values.

Descriptive features of the study population, number of participants, and frequencies of the main variables (gender, age, educational level, skin type, and current sunbed use) are reported for each participating country (Table 1 and Table 2).

The majority of individuals were aged over 35 years (65.5%), while 26.3% were aged 20–35 years and 8.2% were younger than 20 years. Most participants were female (67%). Regarding educational attainment, 65.2% of participants had a high level of education. In terms of skin type, the largest proportion of participants had darker skin types (Type III–IV), accounting for 73% of the sample (31% Type III and 42% Type IV).

**Figure 1:**
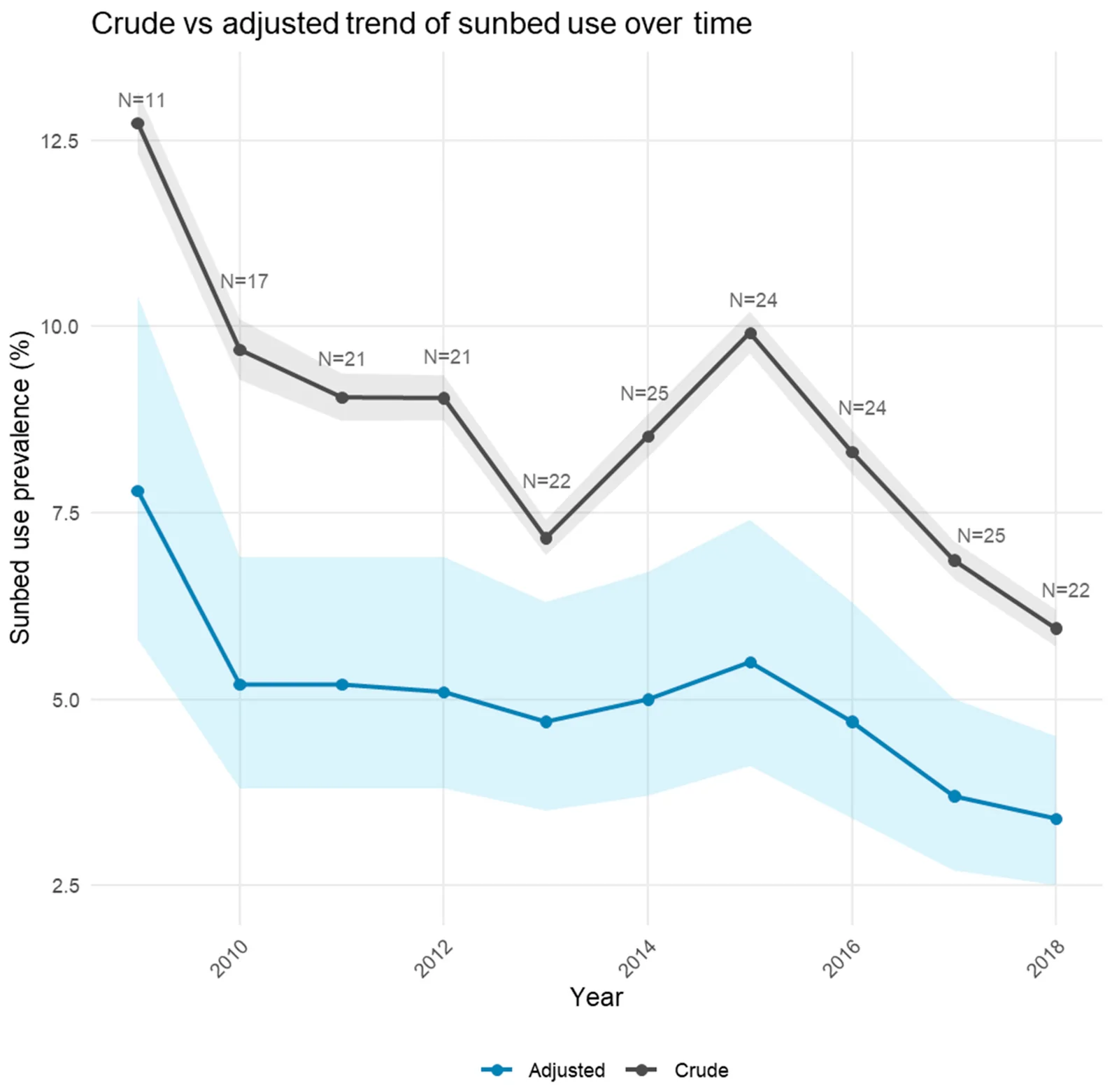
Trends in crude and adjusted prevalence of current sunbed use with 95% confidence intervals, 2009–2018. N denotes the number of participating countries contributing data in each calendar year.

Sunbed use declined substantially over time, as shown by the crude and adjusted prevalence estimates (Figure 1). Crude prevalence decreased from approximately 12.8% in 2009 to 6.0% in 2018, with a consistent downward trajectory despite some fluctuations between 2012 and 2015. The adjusted prevalence, accounting for age, gender, education, skin type, and country, showed a more consistent and gradual decline over time (Figure 1, adjusted). The attenuation of year-to-year fluctuations of adjusted estimates suggests that part of the variability observed in crude estimates may be attributable to changes in the demographic composition of survey participants across survey years. The number of contributing countries increased over time, which may partly explain the variability observed in crude estimates.

Country-specific data checks did not identify evidence of data extraction issues or denominator inconsistencies that would underlie the marked year-to-year fluctuations. In the model including survey year as a continuous variable, the odds of current sunbed use were approximately 6% lower per calendar year (OR = 0.94; 95%CI 0.93, 0.95) (Table 3A).

The multivariable model, including an interaction between year and gender, did not showa significant interaction. However, the interaction terms indicated a significantly steeper temporal decline among younger participants compared with those aged >35 years (interaction OR 0.98, 95% CI 0.96–1.00 for <20 years and 0.96, 95% CI 0.95–0.97 for 20–35 years). The temporal trend was also confirmed by the sensitivity analysis, which retained observations with missing characteristics (using an additional variable layer to capture their missingness; see Table 3B).

**Figure 2:**
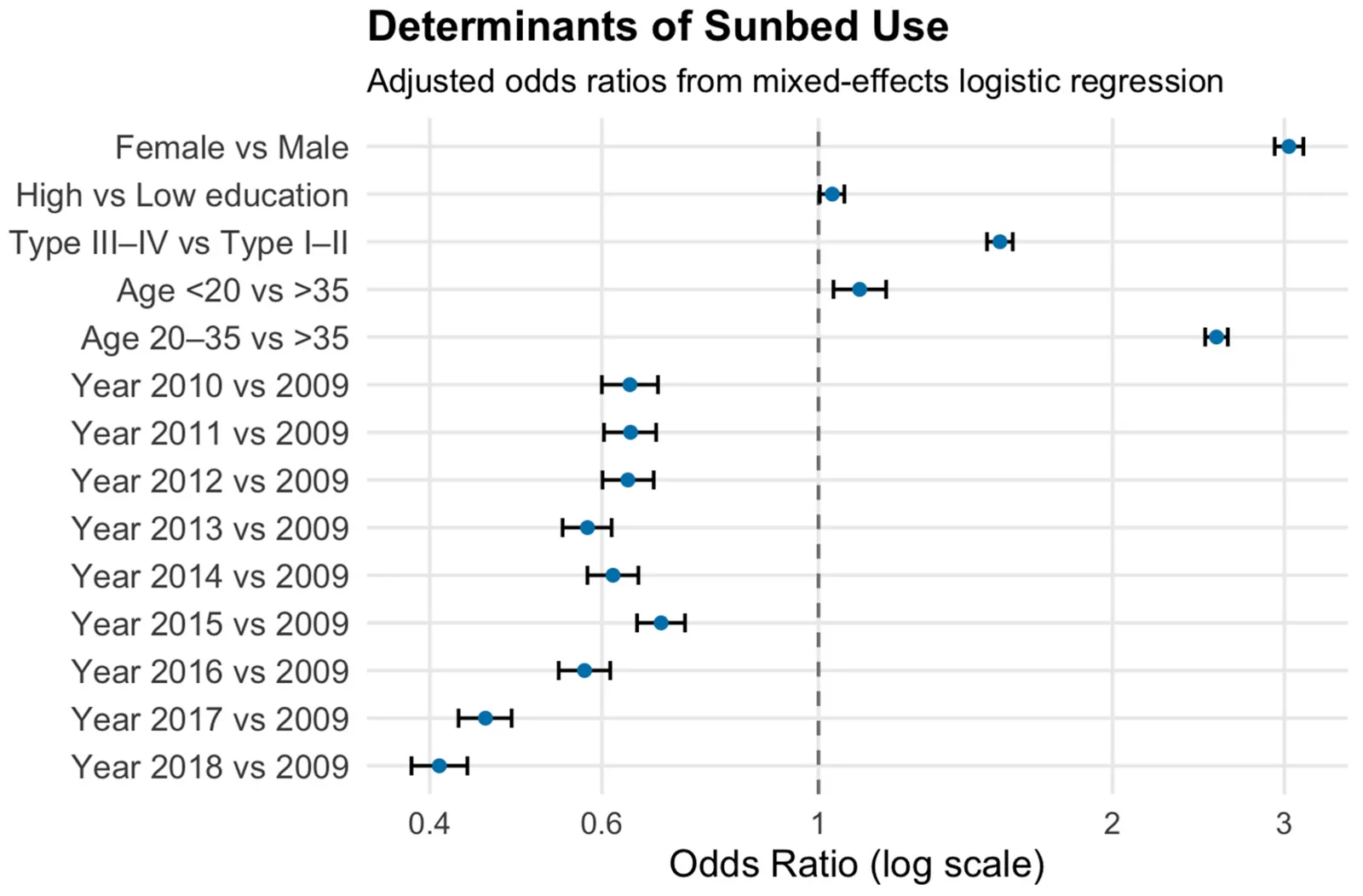
Adjusted odds ratios (ORs) between individual-level characteristics, calendar time, with current sunbed use. ORs and their 95% confidence intervals were obtained from a mixed-effects logistic regression model, including gender, education level, skin phototype, age category, Euromelanoma survey year as categorical fixed effects, and a random intercept for country. Estimates are displayed on a logarithmic scale; the vertical reference line indicates OR = 1.

In mixed-effects logistic regression, substantial between-country heterogeneity persisted. Sunbed use was most strongly associated with female gender (Figure 2; OR 3.01; 95% CI, 2.91–3.12). Younger age showed a pronounced effect: individuals aged 20–35 years had more than twice the odds compared with those >35 (OR 2.54, 2.47–2.61), while adolescents showed a modest increase (OR 1.10, 1.04–1.17). Darker phototypes were associated with higher odds of sunbed use (ORs 1.52 and 1.47–1.56). Education showed only a marginal effect (OR 1.03, 1.00–1.06). Although statistically significant, the effect size was minimal and of limited practical relevance. A strong temporal gradient was observed: odds of sunbed use declined over the study period (OR = 0.94). Substantial between-country variability in sunbed use was observed, as indicated by the random intercept variance (0.81, SE = 0.21).

Some country-specific year-to-year fluctuations, such as that observed in Sweden around 2015, should therefore be interpreted cautiously. Euromelanoma participants constitute a voluntary screening population rather than representative samples of the population collected annually. Changes over the years in campaign communication, recruitment strategies, participating centers, geographic coverage, and participant characteristics may alter sample composition and contribute to short-term fluctuations in reported indoor tanning prevalence. Indeed, previous Euromelanoma experience has shown that changes in campaign messaging and targeting can substantially modify the demographic profile of screening participants [19]. These factors cannot be disentangled retrospectively, and such fluctuations should therefore not necessarily be interpreted as corresponding changes in indoor tanning prevalence in the underlying national population.

Country-specific crude prevalence of sunbed use is presented in Supplementary Figure S2A–D.

To further explore the relationship between geographic location and indoor tanning, country-specific prevalence estimates were plotted against latitude and modeled using LOESS smoothing for each survey year. The interactive visualizations are publicly available at: https://auroragaeta.shinyapps.io/app_sun/ (accessed on 26 August 2026). The LOESS curves showed a broadly positive association between latitude and country-level sunbed-use prevalence across survey years. The increasing number of participating countries in later years provided broader geographical coverage and greater precision around the country-specific estimates, but did not necessarily indicate a strengthening of the latitude–prevalence association over time. The broader geographic coverage and larger number of observations likely allowed a clearer visualization of the relationship between latitude and indoor tanning behavior.

## 4. Discussion

In this large pan-European study, we observed an overall decline in current sunbed use across Europe between 2009 and 2018. Age- and gender-related differences and substantial geographic heterogeneity persisted despite the overall decline, with higher prevalence among women and young adults. Differences in prevalence across countries suggest residual between-country heterogeneity, which may reflect differences in cultural contexts, regulatory environments, or social norms [7]. These results are in line with previous findings from Euromelanoma campaigns [9,19] and other national campaigns [18,21] and provide comparable multiyear data across European countries. The present findings complement our previous Euromelanoma analysis, which focused on ever sunbed use and its determinants, by providing a ten-year description of changes in current sunbed use and differences in temporal trends across population groups and countries.

A substantial temporal decline in current sunbed use was observed during the study period. Compared with the reference year (2009), prevalence decreased progressively in later years. This finding is consistent with international evidence from the post-IARC regulatory era. A systematic review and meta-analysis by Rodriguez-Acevedo et al. (2020) reported a clear decline in indoor tanning prevalence both internationally and across Europe [22]. Similarly, the German National Cancer Aid Monitoring study [21] reported a decline in tanning-bed use between 2012 and 2018. In our study, crude prevalence decreased from approximately 12.8% in 2009 to 6.0% in 2018, while the multivariable model estimated an approximately 6% reduction in the odds of current sunbed use per calendar year.

The persistence of the declining trend after adjustment for gender, age, education, and skin phototype indicates that the observed temporal pattern was not fully explained by changes in participants’ measured characteristics across survey years. The observed decline may reflect changes in sunbed use occurring in the context of other time-varying factors not captured in the dataset, including changes in social norms, access to tanning facilities, and the regulatory and prevention context.

Female gender was strongly associated with sunbed use, with women showing more than threefold higher odds than men, consistent with previous studies [12,16,17,25].

In a French national survey, being female was strongly associated with ever using a sunbed (OR = 3.9) after adjusting for other factors [16]. The marked excess among women may reflect the greater role of appearance-related motivations and social norms surrounding tanning, as described in previous studies. This pattern suggests that prevention interventions may need to address appearance-related motivations and social norms, particularly among young women.

Individuals with higher educational attainment had slightly higher odds of current sunbed use than those with lower educational attainment. Similar associations have been reported in Italy [18], Germany [12,21], and Denmark [13]. This association may reflect higher disposable income, greater use of aesthetic services, or social norms that favor a tanned appearance among more educated groups.

The steeper decline among younger participants is encouraging, but the consistently higher level of use among 20–35 year olds indicates that this age group remains a priority target for prevention efforts. This finding is consistent with previous studies showing that indoor tanning is most common among young adults [7]. As exposure at younger ages is associated with a substantially increased lifetime risk of skin cancer [7,8,25,26], this age group represents a particularly important target for prevention strategies.

Individuals with darker skin types had higher odds of current sunbed use than those with lighter skin types. Similar associations have been reported in a narrative review [19], an Austrian survey [17], an Italian cancer-league campaign [18], and German population studies [14,21]. However, the reasons for this association remain uncertain and cannot be directly assessed in the present dataset, which did not collect information on motivations for tanning, perceived susceptibility, ancestry, or migration background.

As in previous studies, current sunbed use tended to be higher in northern European countries, where natural sunlight is less abundant [9,10,19]. However, factors such as sociocultural norms, commercial availability of tanning salons, or national regulations may also contribute to the observed geographic variation. For example, Italy has relatively high sunbed use despite abundant natural sunlight [19]. The ecological analyses were conducted for descriptive purposes only and should not be interpreted as evidence of causal associations because of the risk of ecological fallacy.

The persistence of the downward trend after adjustment for measured participant characteristics suggests that the observed temporal pattern was not fully explained by differences in age, gender, education and skin phototype across survey years (Figure 1). The attenuation of year-to-year fluctuations after adjustment for confounders suggests that some of the variability in the crude estimates may be attributable to changes in the demographic composition of survey participants across survey years, particularly in terms of age, gender, educational attainment, and skin phototype. Because these characteristics were not equally distributed across countries and years, changes in the composition of the campaign participants may have contributed to fluctuations in crude prevalence estimates.

This study has several strengths. First, it draws on a large multinational dataset comprising more than 340,000 Euromelanoma participants from 33 countries, allowing the assessment of temporal patterns across a broad range of European settings. Second, the repeated cross-sectional design provides a ten-year perspective on changes in current sunbed use. Third, the use of mixed-effects models allowed us to account for clustering and substantial heterogeneity between countries while adjusting for important individual-level characteristics. Fourth, the combination of crude and adjusted prevalence estimates, age-by-time interaction analyses, country-specific trend analyses, and ecological exploration of latitude provides a multidimensional assessment of temporal and geographical patterns.

Several limitations should be acknowledged. The estimates describe individuals attending Euromelanoma screening campaigns and cannot be assumed to represent the general European population. Despite the large overall number of participants, geographical coverage and sample size varied across countries and over time, potentially affecting comparability. As shown by the population characteristics, country-specific shifts in the sample composition were observed over time.

The study period ended in 2018 because comparable Euromelanoma data for subsequent years were unavailable for the present analysis. A new questionnaire was proposed in 2019, and Euromelanoma campaign-based data collection was disrupted by the COVID-19 pandemic [23]. This limits the present analysis’s ability to assess whether the declining trend observed up to 2018 was maintained in the following years. Importantly, the results should therefore be interpreted as describing temporal patterns during the pre-pandemic period and should not be extrapolated to the post-2018 period. Nevertheless, the ten-year observation window provides a substantial period for assessing changes in sunbed use and differences in temporal trends across population groups and countries.

Current sunbed use was assessed using a self-reported question (‘Do you use solarium?’) without a specified recall period; therefore, the temporal recency of reported use cannot be precisely established. However, this measure remains subject to limitations inherent to self-reported behaviors, including potential misclassification and reporting bias. A composite measure combining current and past use was not used because the objective of this study was to assess changes in current sunbed use over time, whereas lifetime/ever use had been examined previously in the Euromelanoma dataset [19].

Participants in the Euromelanoma campaigns may also have been more aware of melanoma risk factors than the general population, limiting the generalisability of the findings. The use of adjusted regression models accounted for differences in measured participant characteristics across years and countries, but did not eliminate potential bias related to opportunistic sampling. Approximately 16% of observations were excluded from the multivariable analyses because of missing data. Although complete-case analysis ensured a common analytical sample across models, differential missingness between countries may have introduced selection bias.

Because Euromelanoma participants constitute a voluntary screening population rather than annually repeated representative samples, temporal and geographical comparisons may be influenced by changes in campaign recruitment, participating centres, geographical coverage, and participant characteristics. Adjustment for measured individual-level characteristics does not eliminate potential bias arising from unmeasured differences in recruitment or awareness.

Country sample sizes were highly unbalanced, with some countries contributing relatively few participants and others contributing very large samples. Although the mixed-effects framework accounted for clustering by country, estimates and overall model-based predictions may still have been influenced more strongly by countries with larger contributions, while estimates for countries with small samples are subject to greater uncertainty.

Country-specific random effects should not be interpreted as causal country effects. National characteristics such as climate, sunbed legislation and enforcement, and the composition of the screening population are likely to differ systematically across settings. The random intercept was included to account for the clustering of participants within countries and to quantify residual between-country heterogeneity, rather than to estimate the effect of any specific national characteristic. Moreover, because random effects are shrunk towards the overall mean, estimates for countries contributing few participants should be interpreted with particular caution.

Finally, although the mixed-effects approach accounted for between-country variability, unmeasured country-level factors such as regulatory environments, cultural norms, or industry practices may also contribute to differences in sunbed use across countries.

## 5. Conclusions

Current sunbed use among Euromelanoma campaign participants declined substantially between 2009 and 2018, with persistent differences by age, gender, and country. These findings provide evidence of a sustained pre-pandemic decline, although continued surveillance is needed to determine whether these patterns have persisted in subsequent years. The findings are consistent with a broader decline observed during a period of evolving prevention and regulatory measures, although the observational design does not allow attribution of the temporal trend to specific policies. Young adults and women remained the groups with the highest levels of use, supporting their continued prioritization in prevention efforts. Sustained surveillance and context-specific prevention strategies remain warranted, particularly in countries where sunbed use remains comparatively high.

## Figures and Tables

**Table 1:** Descriptive features of the study population. Relative frequencies of the main variables (gender, age) are reported for each participating country. * taking as denominator the sum of non-missing observations. ** more than 10% was missing.^1^ sum of non-missing observations.

	Sunbed Use *		Gender *		Age Category *		
Characteristic	Yes	No	Female	Male	<20	20–35	>35
Country	N = 32,910 ^1^	N = 363,865 ^1^	N = 2,723,931	N = 1,332,831 ^1^	N = 32,119 ^1^	N = 103,090 ^1^	N = 256,783 ^1^
Armenia	46 (6%)	697 (94%)	404 (54%)	340 (46%)	81 (11%)	175 (23%)	492 (66%)
Belarus	54 (11%)	437 (89%)	387 (77%)	114 (23%)	56 (11%)	209 (42%)	236 (47%)
Belgium	2943 (19%)	12,877 (81%)	9858 (62%)	6034 (38%)	389 (2%)	4307 (27%)	11,334 (71%)
Bosnia and Herzegovina	630 (5%)	12,303 (95%)	8591 (64%)	4861 (36%)	1045 (8%)	4052 (30%)	8279 (62%)
Croatia	681 (8%)	8293 (92%)	5894 (62%)	3565 (38%)	924 (10%)	2118 (22%)	6441 (68%)
Cyprus	2 (3%)	56 (97%)	28 (56%) **	22 (44%) **	4 (9%) **	13 (29%) **	28 (62%) **
Czech Republic	1475 (9%)	15,442 (91%)	11,334 (67%)	5645 (33%)	1700 (10%)	4801 (28%)	10,485 (62%)
Denmark	432 (9%)	4179 (91%)	2879 (60%)	1941 (40%)	207 (4%)	929 (20%)	3592 (76%)
Estonia	286 (14%)	1722 (86%)	1497 (73%)	551 (27%)	255 (12%)	449 (22%)	1351 (66%)
Finland	125 (6%)	1880 (94%)	1276 (63%)	741 (37%)	35 (2%)	459 (23%)	1536 (76%)
Georgia	88 (2%)	4746 (98%)	3702 (76%)	1155 (24%)	379 (8%)	997 (21%)	3424 (71%)
Germany	1230 (10%)	11,617 (90%)	7915 (62%)	4784 (38%)	295 (2%)	1843 (15%)	10,151 (83%)
Greece	1209 (3.0%)	39,244 (97%)	26,035 (64%)	14,464 (36%)	3566 (9%)	14,351 (36%)	21,970 (55%)
Hungary	4213 (19%)	18,469 (81%)	16,649 (70%)	7106 (30%)	1654 (7%)	6943 (29%)	15,075 (64%)
Ireland	83 (7%) **	1063 (93%) **	905 (67%)	454 (33%)	43 (3%)	215 (16%)	1056 (80%)
Italy	895 (22%)	3120 (78%)	2682 (63%)	1577 (37%)	425 (10%)	993 (23%)	2836 (67%)
Latvia	568 (24%)	1806 (76%)	1865 (79%)	508 (21%)	206 (9%)	726 (31%)	1439 (61%)
Lithuania	846 (13%)	5609 (87%)	4621 (72%)	1838 (28%)	576 (9%)	1613 (25%)	4194 (66%)
Malta	8 (1%)	673 (99%)	408 (58%)	295 (42%)	78 (11%)	101 (14%)	520 (74%)
Moldova	2 (4%)	54 (96%)	39 (70%)	17 (30%)	8 (14%)	10 (18%)	38 (68%)
North Macedonia	71 (4%)	1934 (96%)	1246 (63%)	718 (37%)	131 (7%)	511 (27%)	1241 (66%)
Norway	902 (19%)	3750 (81%)	2930 (62%)	1808 (38%)	58 (1%)	1129 (24%)	3487 (75%)
Poland	2793 (13%)	18,569 (87%)	14,636 (66%)	7598 (34%)	2570 (12%)	7402 (33%)	12,234 (55%)
Portugal	269 (2%)	13,497 (98%)	8530 (62%)	5136 (38%)	575 (5%)	2728 (22%)	9292 (74%)
Romania	575 (5%)	11,901 (95%)	7755 (61%)	4965 (39%)	3998 (31%)	2535 (20%)	6246 (49%)
Russia	4469 (8%)	48,559 (92%)	44,678 (81%)	10,615 (19%)	5346 (10%)	18,113 (33%)	30,905 (57%)
Serbia	1264 (7%)	17,444 (93%)	12,426 (65%)	6592 (35%)	1187 (6%)	4049 (22%)	13,499 (72%)
Slovenia	56 (4%)	1227 (96%)	636 (49%)	668 (51%)	48 (4%)	243 (19%)	1006 (78%)
Spain	1352 (8%)	15,202 (92%)	10,900 (66%)	5659 (34%)	323 (5%) **	2081 (29%) **	4685 (66%) **
Sweden	2892 (9%)	28,533 (91%)	21,344 (65%)	11,501 (35%)	705 (2%)	4854 (15%)	27,361 (83%)
Switzerland	1730 (6%)	27,038 (94%)	17,007 (58%)	12,276 (42%)	1174 (5%)	6020 (21%)	21,538 (75%)
Turkey	25 (1%)	2862 (99%)	1922 (65%)	1040 (35%)	305 (10%)	1091 (37%)	1557 (53%)
Ukraine	696 (2%)	29,062 (98%)	21,414 (71%)	8695 (29%)	3773 (13%)	7030 (23%)	19,255 (64%)

N (%) presented in each box, unless otherwise stated.

**Table 2:** Descriptive features of the study population. The number of relative frequencies of the main variables (educational level, skin type, and current sunbed use) are reported for each participating country. * taking as denominator the sum of non-missing observation. ** more than 10% was missing. ^1^ sum of non-missing observations.

	Education *		Skin Type *			
Characteristic	High	Low	Type I	Type II	Type III	Type IV
Country	N = 249,538 ^1^	N = 133,153 ^1^	N = 27,265 ^1^	N = 83,109 ^1^	N = 124,812 ^1^	N = 165,845 ^1^
Armenia	554 (79%)	148 (21%)	133 (18%)	169 (23%)	183 (25%)	245 (34%)
Belarus	408 (85%)	70 (15%)	31 (7%)	84 (18%)	154 (32%)	211 (44%)
Belgium	10,047 (66%)	5225 (34%)	924 (6%)	4831 (30%)	2939 (18%)	7241 (45%)
Bosnia and Herzegovina	6868 (52%)	6270 (48%)	975 (7%)	2385 (18%)	4870 (37%)	5003 (38%)
Croatia	3872 (42%)	5352 (58%)	368 (4%)	1383 (15%)	3999 (43%)	3652 (39%)
Cyprus	28 (48%)	30 (52%)	8 (14%)	23 (40%)	16 (28%)	10 (18%)
Czech Republic	2458 (27%) **	6753 (73%) **	796 (5%)	3291 (20%)	6000 (36%)	6594 (40%)
Denmark	3195 (68%)	1493 (32%)	235 (5%)	702 (15%)	1677 (36%)	2102 (45%)
Estonia	1272 (66%)	654 (34%)	127 (6%)	931 (46%)	213 (11%)	741 (37%)
Finland	1444 (74%)	515 (26%)	81 (4%)	394 (20%)	726 (36%)	791 (40%)
Georgia	3903 (81%)	916 (19%)	655 (14%)	487 (10%)	1723 (36%)	1974 (41%)
Germany	4634 (54%) **	3913 (46%) **	608 (5%)	2371 (19%)	3469 (28%)	6070 (48%)
Greece	29,828 (74%)	10,449 (26%)	2008 (5%)	10,340 (26%)	14,312 (35%)	13,694 (34%)
Hungary	13,057 (56%)	10,151 (44%)	1383 (6%)	4293 (18%)	7006 (30%)	10,757 (46%)
Ireland	831 (63%)	490 (37%)	246 (18%)	209 (16%)	389 (29%)	486 (37%)
Italy	1412 (35%)	2648 (65%)	224 (5%)	861 (21%)	1242 (30%)	1859 (44%)
Latvia	1819 (78%)	509 (22%)	143 (6%)	1255 (54%)	169 (7.2%)	768 (33%)
Lithuania	5071 (80%)	1282 (20%)	460 (7%)	1278 (20%)	1968 (30%)	2753 (43%)
Malta	269 (40%)	410 (60%)	52 (7%)	259 (37%)	91 (13%)	297 (42%)
Moldova	42 (76%)	13 (24%)	0 (0%)	13 (23%)	19 (34%)	24 (43%)
North Macedonia	1045 (54%)	900 (46%)	282 (14%)	463 (23%)	642 (32%)	617 (31%)
Norway	3825 (81%)	877 (19%)	116 (3%)	751 (16%)	1431 (30%)	2415 (51%)
Poland	14,230 (66%) **	7259 (34%) **	1597 (7%)	3938 (18%)	8084 (36%)	8791 (39%)
Portugal	5692 (46%)	6666 (54%)	928 (7%)	5431 (40%)	823 (6.0%)	6461 (47%)
Romania	6118 (50%)	6043 (50%)	791 (6%)	2990 (23%)	4499 (35%)	4479 (35%)
Russia	40,421 (76%)	13,062 (24%)	6498 (12%)	9521 (18%)	16,366 (30%)	21,569 (40%)
Serbia	8818 (47%)	9982 (53%)	1123 (6%)	3612 (19%)	6058 (32%)	8276 (43%)
Slovenia	766 (59%)	536 (41%)	46 (4%)	203 (16%)	553 (42%)	500 (38%)
Spain	12,151 (73%)	4382 (27%)	1894 (12%)	4571 (28%)	3376 (21%)	6610 (40%)
Sweden	22,520 (69%)	10,308 (31%)	927 (2.8%)	5643 (17%)	8295 (25%)	17,679 (54%)
Switzerland	20,102 (72%)	7932 (28%)	1377 (4.8%)	5962 (21%)	8079 (28%)	13,077 (46%)
Turkey	1715 (59%)	1186 (41%)	517 (18%)	625 (22%)	656 (23%)	1093 (38%)
Ukraine	21,123 (76%)	6729 (24%)	1712 (5.8%)	3840 (13%)	14,785 (50%)	9006 (31%)

N (%) presented in each box, unless otherwise stated.

**Table 3:** (**A**) Multivariable mixed-effects logistic regression models for current sunbed use: main effects, gender by year interaction, and age × year interaction. (**B**) Sensitivity analyses using missing values as an additional category in multivariable mixed-effects logistic regression models.

**(A)**					
		**OR**	**Low 95% CI**	**Up 95% CI**	***p*** **-Value**
No interaction	Gender: Female vs. male	3.01	2.91	3.12	<0.001
	Educational level: High vs. Low	1.03	1.00	1.06	0.02
	Skin type: III-IV vs. I-II	1.52	1.47	1.56	<0.001
	Age: <20 vs. >35	1.10	1.04	1.17	<0.001
	Age: 20–35 vs. >35	2.54	2.47	2.61	<0.001
	Year (continuous)	0.939	0.938	0.94	<0.001
Gender by time interaction	Gender: Female vs. male	3.31	2.83	3.87	<0.001
	Educational level: High vs. Low	1.03	1.00	1.06	0.02219
	Skin type: III–IV vs. I–II	1.52	1.47	1.56	<0.001
	Age: <20 vs. >35	1.10	1.04	1.17	0.002
	Age: 20–35 vs. >35	2.54	2.47	2.61	<0.001
	Year (continuous)	0.94	0.93	0.95	<0.001
	Interaction: Gender by year	0.99	0.98	1.00	0.232
Age by time interaction	Gender: Female vs. male	3.02	2.92	3.12	<0.001
	Educational level: High vs. Low	1.03	1.00	1.06	0.03
	Skin type: III–IV vs. I–II	1.52	1.48	1.57	<0.001
	Age: <20 vs. >35	1.52	1.15	2.01	<0.001
	Age: 20–35 vs. >35	4.36	3.83	4.97	<0.001
	Year (continuous)	0.96	0.95	0.96	<0.001
	Interaction Age by Year: age <20 vs. >35	0.98	0.96	1.00	0.02
	Interaction Age by Year: age 20–35 vs. >35	0.96	0.95	0.97	<0.001
(**B**)					
	**Variable**	**OR**	**95%Low CI**	**95%up CI**	***p*** **-Value**
	Gender Female vs. Male	2.90	2.81	2.99	<0.001
Model without interaction	Gender Missing vs. Male	2.20	1.96	2.45	<0.001
	Education level High vs. Low	1.04	1.01	1.07	0.01
	Education level Missing vs. Low	0.85	0.80	0.9	<0.001
	Skin type: III–IV vs. I–II	1.50	1.46	1.55	<0.001
	Skin type: Missing vs. I–II	0.91	0.82	1.00	0.06
	Age: <20 vs. >35	0.97	0.92	1.03	0.4
	Age: 20–35 vs. >35	2.58	2.51	2.64	<0.001
	Age: Missing vs. >35	1.38	1.29	1.48	<0.001
	Year continuous	0.94	0.93	0.94	<0.001
Model with gender by year interaction	Gender Female vs. Male	3.56	3.09	4.10	<0.001
	Gender Missing vs. Male	1.73	1.25	2.41	<0.001
	Education level High vs. Low	1.04	1.01	1.07	0.01
	Education level Missing vs. Low	0.85	0.80	0.90	<0.001
	Skin type: III–IV vs. I–II	1.50	1.46	1.55	<0.001
	Skin type: Missing vs. I–II	0.91	0.82	1.00	0.06
	Age: <20 vs. >35	0.98	0.92	1.03	0.4
	Age: 20–35 vs. >35	2.58	2.51	2.64	<0.001
	Age: Missing vs. >35	1.39	1.29	1.49	<0.001
	Year continuous	0.95	0.94	0.96	<0.001
	Interaction Gender by Year: Female	0.98	0.97	1.00	<0.001
	Interaction: Gender by Year: Missing: Year	1.02	0.99	1.04	0.17
Model with age by year interaction	Gender Female vs. Male	2.90	2.81	2.99	<0.001
	Gender Missing vs. Male	2.17	1.95	2.43	<0.001
	Education level High vs. Low	1.04	1.01	1.07	0.01
	Education level Missing vs. Low	0.85	0.80	0.90	<0.001
	Skin type: III–IV vs. I–II	1.51	1.46	1.55	<0.001
	Skin type: Missing vs. I–II	0.91	0.82	1.00	0.05
	Age: <20 vs. >35	1.44	1.16	1.79	<0.001
	Age: 20–35 vs. >35	4.37	3.88	4.91	<0.001
	Age: Missing vs. >35	3.45	2.66	4.49	<0.001
	Year continuous	0.96	0.95	0.96	<0.001
	Interaction Age by Year: <20 vs. >35	0.97	0.96	0.99	<0.001
	Interaction Age by Year: 20–35 vs. >35	0.96	0.95	0.97	<0.001
	Interaction Age by Year: Missing vs. >35	0.94	0.92	0.95	<0.001

## Data Availability

The data that support the findings of this study are available from the corresponding author upon reasonable request.

## Supplementary Materials

The following supporting information can be downloaded at: https://www.mdpi.com/article/10.3390/cancers18213548/s1, Figure S1, Countries participation and number of participants across years. Figure S2A, Temporal trends in current sunbed use from 2009 to 2018 by country and gender. Figure S2B, Temporal trends in current sunbed use from 2009 to 2018 by country and age group. Figure S2C Temporal trends in current sunbed use from 2009 to 2018 by country and education (high vs. low). Figure S2D, Temporal trends in current sunbed use from 2009 to 2018 by country and skin type I–II vs. III–IV).
