# Supplementary material for: Indoor Tanning in Europe: Temporal Trends, Sociodemographic Determinants from the Euromelanoma Campaign (2009–2018)
Source: Cancers (Basel). 2026 Nov 4;18(21):3548. doi: 10.3390/cancers18213548 (PMC13761959; doi:10.3390/cancers18213548)

Supplementary material

Figure S1, Countries participation and number of participants across years, full sample 410 937 participants.

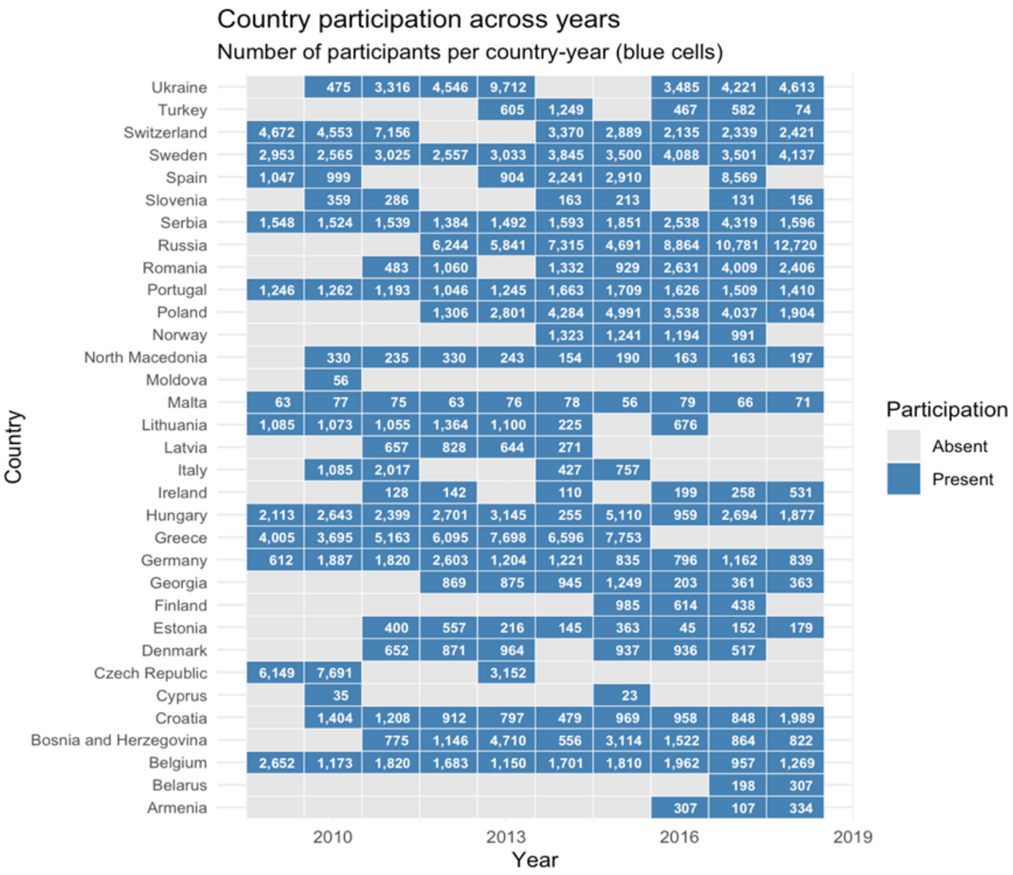

Figure S2A, Temporal trends in current sunbed use from 2009 to 2018 by country and gender.

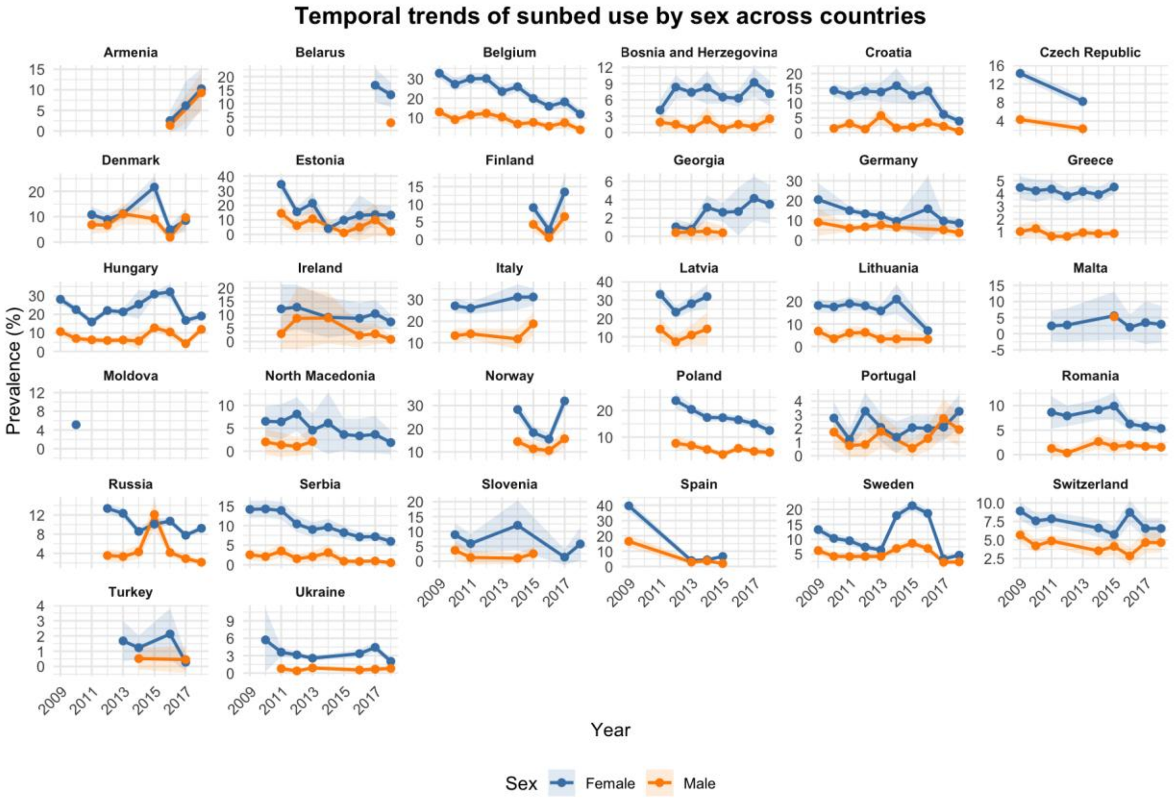

Figure S2B, Temporal trends in current sunbed use from 2009 to 2018 by country and age group.

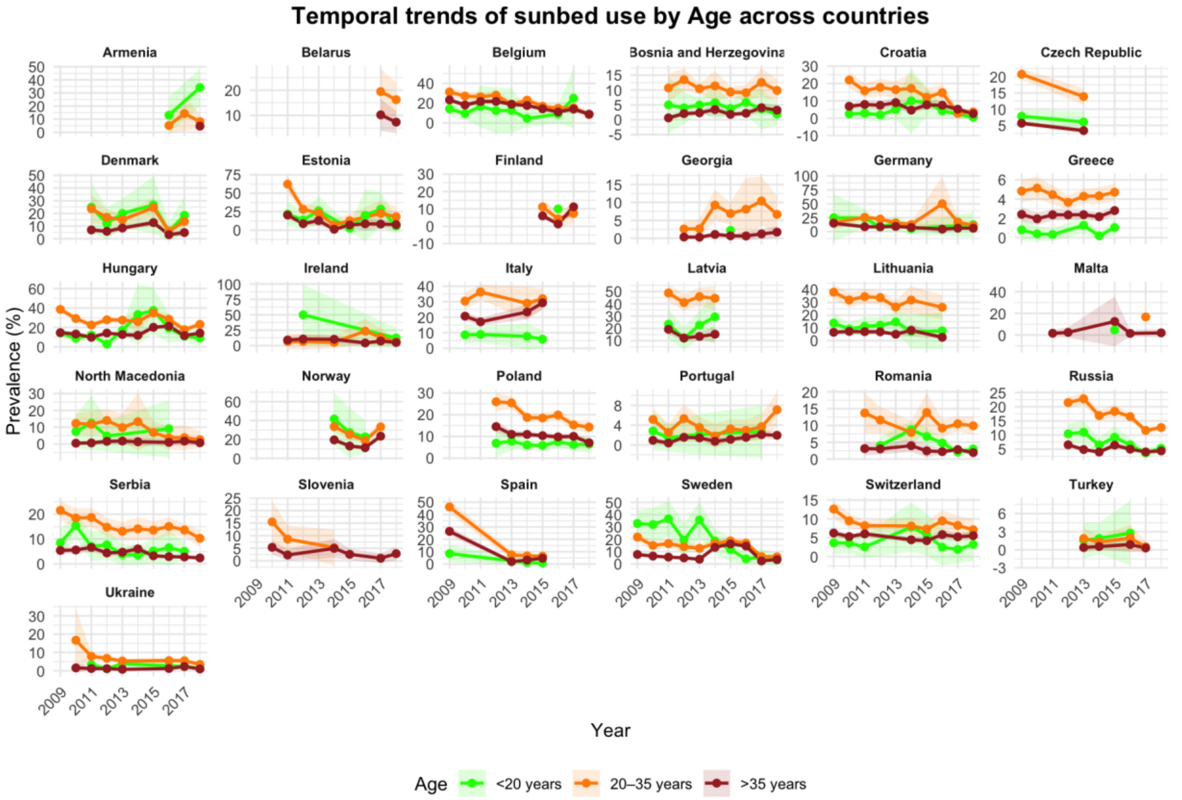

Figure S2C Temporal trends in current sunbed use from 2009 to 2018 by country and education (high vs low).

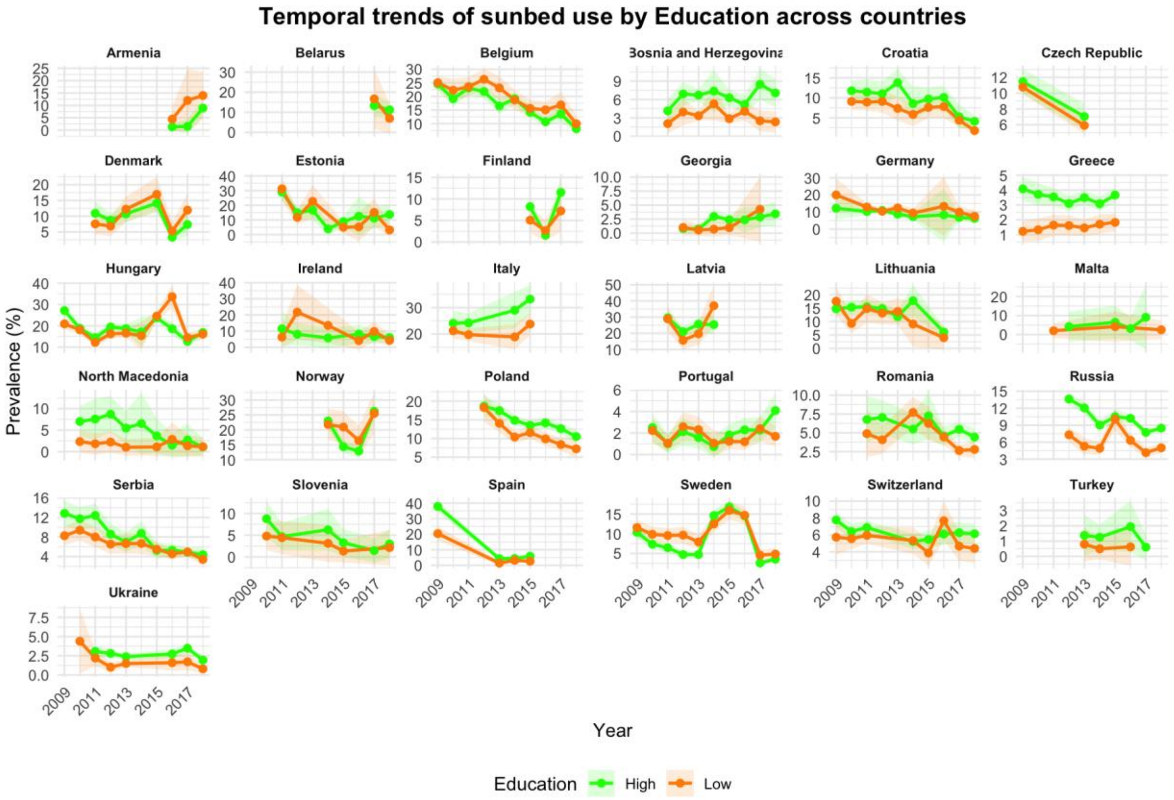

Figure S2D, Temporal trends in current sunbed use from 2009 to 2018 by country and skin type I-II vs III-IV).

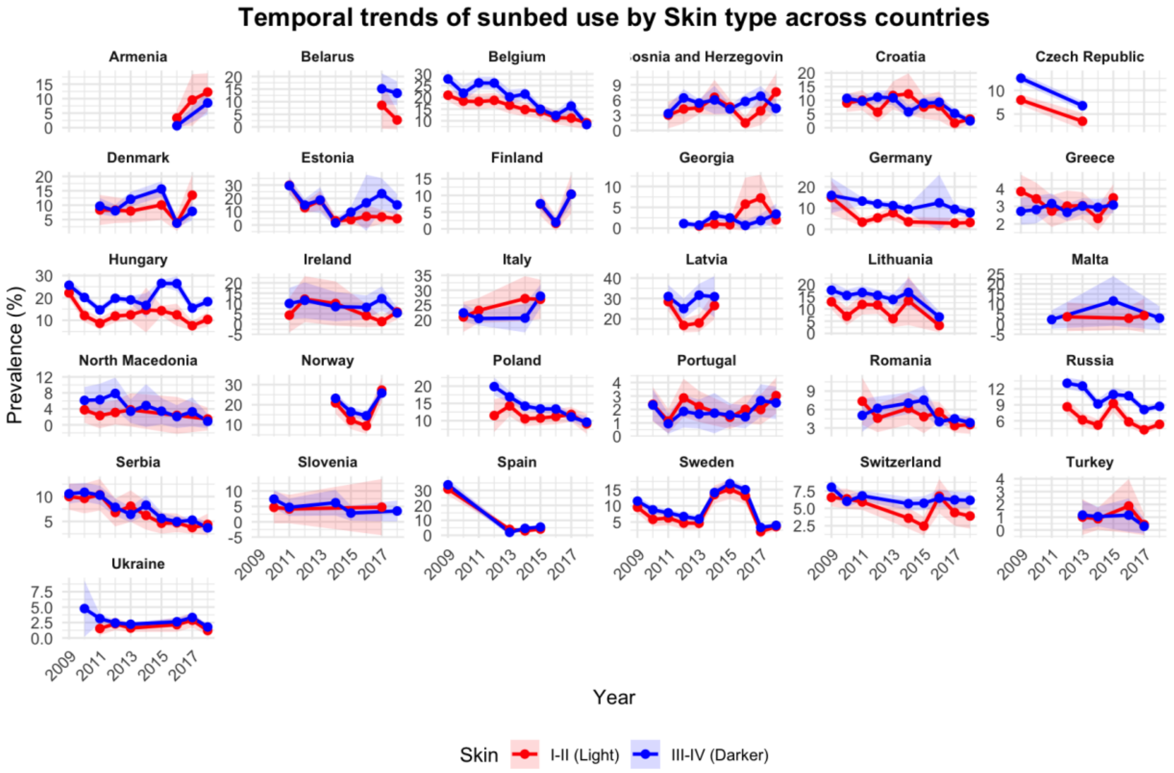

Supplement: Supplementary file 1 [file cancers-18-03548-s001.zip › cancers-4644186-supplementary.pdf]
